# Clinical Efficacy of Different Therapies for Painful Shoulder Conditions: A Network Meta-Analysis of Randomized Controlled Trials

**DOI:** 10.3390/healthcare13222920

**Published:** 2025-11-14

**Authors:** Kuan-Han Chen, Sih-Yu Guo, Hung-Cheng Chen, Chiu-Yueh Yang

**Affiliations:** 1 Emergency Room, NewYork-Presbyterian Brooklyn Methodist Hospital, Brooklyn, New York, NY 11215, USA; chenkuanhan1997@gmail.com; 2 Operation Room, Department of Nursing, National Taiwan University Hospital, Taipei 100115, Taiwan; sihyu0903@ntuh.gov.tw; 3 Department of Electrical Engineering, National Chin-Yi University of Technology, Taichung 411030, Taiwan; hcchen@ncut.edu.tw; 4 Department of Health Business Administration, Hungkuang University, Taichung 433304, Taiwan

**Keywords:** frozen shoulder, acupuncture, sodium hyaluronate, network meta-analysis

## Abstract

**Objective:** This study aimed to evaluate, through a network meta-analysis, the short- and long-term efficacy of both Western medical therapies and traditional Chinese medical therapy (acupuncture) in improving symptoms of shoulder pain. **Methods:** A comprehensive computer-based search was conducted in Embase, Cochrane Library, Web of Science, and PubMed databases for randomized controlled trials (RCTs) related to Western and Chinese medical treatments for shoulder pain measured by visual analogue scale (VAS) scores. All researchers independently screened and selected studies, extracted data, and assessed the risk of bias. Studies that met quality standards were analyzed using Stata 16.0 and Review Manager 5.4 software. **Results:** A total of 269 articles were retrieved, and 15 were ultimately included in the network meta-analysis, covering nine types of Western and Chinese medical therapies. The total sample size was 1114 cases, with 557 in an experimental group and 557 in a control group. In terms of reducing VAS scores at 4 weeks after treatment, sham acupuncture was significantly less effective than acupuncture (MD: 19.39; 95% CI: 0.66–38.12), indicating that acupuncture had a better short-term effect on pain relief at 4 weeks. In terms of reducing VAS scores at 12 weeks after treatment, sodium hyaluronate (hyaluronate) was more effective than physical therapy (PT) in reducing long-term pain (MD: −19.57; 95% CI: −37.23–−1.90); suprascapular nerve block (SSNB) (MD: −9.11; 95% CI: −16.02–−2.20) and arthroscopic capsular release (MD: −16.07; 95% CI: −30.16–−1.97) were also more effective than PT. The top three treatments in terms of clinical efficacy for painful shoulder conditions were hyaluronate, SSNB, and arthroscopic capsular release. **Conclusions:** For the treatment of shoulder pain, hyaluronate, SSNB, and arthroscopic capsular release showed greater potential long-term efficacy in pain reduction than PT, with hyaluronate showing the best effect.

## 1. Introduction

Shoulder pain is a common clinical condition, and possible causes include frozen shoulder, scapulohumeral periarthritis, adhesive capsulitis, and subacromial impingement syndrome [1]. Clinically, patients present with shoulder pain and a restricted range of motion, which can progressively worsen and even lead to local muscle atrophy [2]. This condition is most common in people aged 40–60 years and is characterized by severe pain and limited movement, significantly affecting quality of life and work efficiency, as well as leading to economic consequences due to pain and stiffness [3,4,5]. Although frozen shoulder or scapulohumeral periarthritis is often self-limiting, its course can last for years, causing persistent pain and distress [6,7]. Therefore, it is necessary to seek treatments that are effective, rapid, and safe for pain relief.

There are various treatment options for shoulder pain, including both Western and traditional Chinese medicine. In Western medicine [8], treatments include PT, rehabilitation, arthroscopic capsular release, and intra-articular corticosteroid injections [9,10,11,12,13]. Conservative, non-surgical treatments are generally preferred, such as traditional PT or exercise therapy. To reduce inflammation and improve pain, medications such as non-steroidal anti-inflammatory drugs (NSAIDs) or corticosteroids are commonly used [14,15,16]. In recent years, platelet-rich plasma (PRP), which is derived from autologous blood with a high concentration of platelets and is rich in cytokines, bioactive mediators, and growth factors, has attracted attention for its potential to promote tissue repair [17,18,19,20,21]. Similarly, hyaluronic acid has anti-inflammatory effects and is known to protect damaged cartilage and improve synovial abnormalities [22,23,24]. These are considered relatively conservative treatment approaches.

More aggressive interventions, such as invasive surgical treatments, are also considered, including SSNB and arthroscopic capsular release. SSNB has been found to be a safe and effective method for managing shoulder pain. Similarly, arthroscopic capsular release is considered for its high safety and efficacy in relieving pain in adhesive capsulitis for patients with frozen shoulder or scapulohumeral periarthritis [25,26,27,28,29].

In contrast, traditional Chinese medicine has also received much attention in the clinical treatment of frozen shoulder or scapulohumeral periarthritis, particularly acupuncture for pain relief [30,31]. Studies have shown that acupuncture can intervene in frozen shoulder or scapulohumeral periarthritis by improving arterial spasm and regulating neural reflexes [32]. Joint mobilization, which combines traditional massage with biomechanics and emphasizes clinician–patient collaboration, has become a popular method for treating frozen shoulder or shoulder pain [33,34]. Besides acupuncture, massage, and the joint mobilization techniques mentioned above, the common treatments for frozen shoulder or shoulder pain in traditional Chinese medicine practice include the use of traditional Chinese medicine external plasters, such as Cheezheng Xiaotong plasters [35] and Gutong plasters [36]. Acupuncture was the only Eastern medicine modality with sufficient RCT data for quantitative synthesis. Therefore, this study focuses solely on the pain-relieving effects of acupuncture within the framework of traditional Chinese medicine treatment. At the current stage, this research does not address the analgesic efficacy of traditional Chinese medicine external application plasters.

Despite the variety of Western and Chinese medical treatments for shoulder pain, the most effective method for pain relief remains uncertain. Furthermore, the effects of different therapies on short- and long-term pain relief have not been clearly established and warrant further investigation [37]. Therefore, this study is the first to simultaneously evaluate different Western and Chinese therapies, dividing pain relief effects into short-term (4 weeks post-treatment) and long-term (12 weeks post-treatment) outcomes, focusing on the efficacy of pain reduction and identifying clinically significant findings.

This study used a network meta-analysis approach to compare and rank the clinical efficacy of commonly used Western medical treatments and acupuncture for shoulder pain, exploring the advantages of each treatment in short- and long-term pain relief, as well as providing evidence-based support for clinical decision-making. We hypothesized that certain interventions, particularly intra-articular corticosteroid injection, intra-articular PRP injection, intra-articular hyaluronic acid injection, SSNB, arthroscopic capsular release, acupuncture, and sham acupuncture, would demonstrate greater potential efficacy in shoulder pain reduction than PT.

## 2. Materials and Methods

The meta-analysis component of this study was conducted in accordance with the Preferred Reporting Items for Systematic Reviews and Meta-Analyses (PRISMA) guidelines and has been registered on Open Science Framework (OSF) (Registration Number: 10.17605/OSF.IO/VQC6W).

### 2.1. Eligibility Criteria

The inclusion criteria consisted of the following: (1) study type: published clinical RCTs in English; (2) participants: patients diagnosed with shoulder pain conditions, including but not limited to frozen shoulder, scapulohumeral periarthritis, adhesive capsulitis, and subacromial impingement syndrome, according to recognized diagnostic criteria with clear efficacy standards; no restrictions on age, race, or gender; (3) interventions: intra-articular corticosteroid injection, intra-articular PRP injection, intra-articular hyaluronic acid injection, SSNB, arthroscopic capsular release, acupuncture, sham acupuncture, and PT; and (4) outcomes: short-term (4-week) and long-term (8–12-week) pain VAS scores, allowing for a quantitative comparison of pain outcomes across studies [38].

The exclusion criteria included the following: (1) non-RCT studies; (2) duplicate publications; (3) inconsistent interventions; (4) outcomes not reported; (5) unreferenced or self-defined diagnostic criteria; (6) incomplete or erroneous data; and (7) severe comorbidities.

### 2.2. Search Methods and Study Selection

A computer-based search was conducted in Embase, Cochrane Library, Web of Science, and PubMed databases. The search terms included scapulohumeral periarthritis, frozen shoulder, adhesive periarthritis of shoulder, periarthritis of shoulder, shoulder pain, shoulder osteodystrophy, shoulder osteoarthritis, shoulder periarthritis, proliferation therapy, prolotherapy, dextrose prolotherapy, blind glucocorticoid injection, hypertonic dextrose injection, corticosteroid injections, glucose prolotherapy, PT, intra-articular injection, acupuncture, needle, and randomized controlled trial. Please refer to the Supplementary Materials for detailed search strategies of all databases. Both subject headings and free-text terms were used, with the search period ranging from database inception to 30 April 2025.

### 2.3. Data Collection

Two researchers independently performed the study selection process according to the screening criteria, extracted the data, and screened each other’s results. If there were any discrepancies, another clinical physician made the final judgment and decision. EndNote 2025 software was used for initial screening and deduplication, followed by title and abstract screening and then a full-text review. If data were incomplete, the original authors were contacted for further information. The extracted data were entered into Excel, including author, publication year, sample size, disease duration, interventions, treatment duration, and outcomes. Outcome data for pain intensity were converted to a 0–100 point scale.

### 2.4. Risk of Bias Assessment

The guidelines of the Cochrane Handbook for Systematic Reviews of Interventions were followed to assess the risk of bias of the included studies [39].

### 2.5. Data Synthesis and Analysis

All outcome data were analyzed using random-effects models. Continuous variables are expressed as mean ± standard deviation. A network meta-analysis was performed using Stata 16.0, with the network command used for data preprocessing, drawing evidence network diagrams, and ranking interventions using the surface under the cumulative ranking curve (SUCRA). The size of the node in the evidence network diagram indicates the number of patients receiving each intervention, and the thickness of the lines between nodes represents the number of studies comparing the two interventions [40,41,42,43,44,45]. SUCRA values are expressed as percentages, with higher values indicating greater efficacy [46,47]. Consistency was assessed using node-splitting models, and funnel plots were used to assess publication bias when more than 10 studies were included for an outcome [48,49]. Review Manager 5.4 was used for the risk of bias assessment.

## 3. Results

### 3.1. Literature Search Results

**Figure 1:**
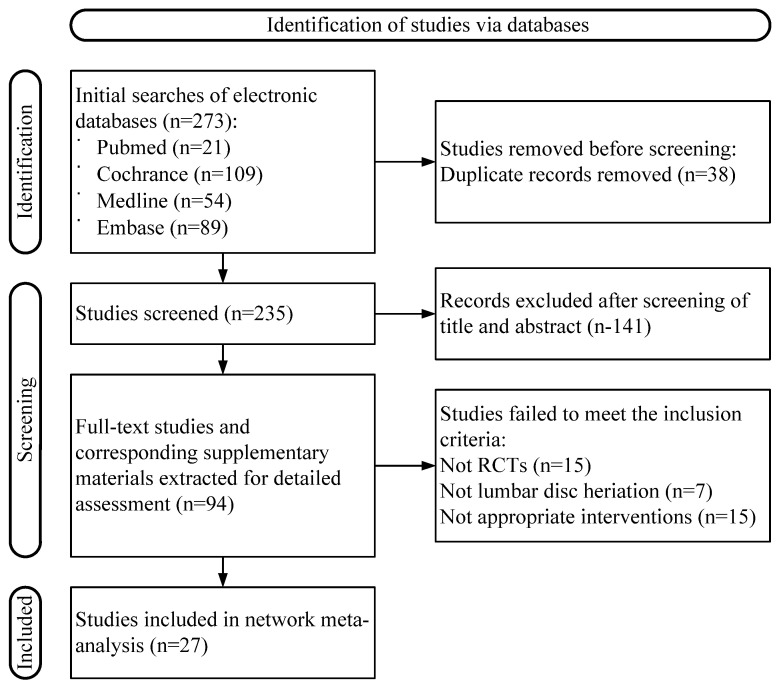
Flowchart of search and screening of the included studies.

A total of 269 articles were identified, and, after screening, 15 were finally included in the network meta-analysis [50,51,52,53,54,55,56,57,58,59,60,61,62,63,64], among which 3 were three-arm clinical trials [54,57,59], and the remaining 12 were double-arm clinical trials. A flowchart of the study selection process is shown in Figure 1. A total of 1114 patients diagnosed with shoulder pain were included, with 557 in the experimental group and 557 in the control group. The basic characteristics of all included studies are presented in Table 1.

### 3.2. Risk of Bias Assessment

**Figure 2:**
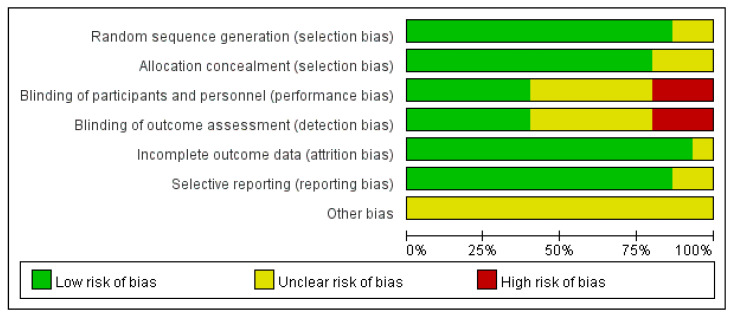
Risk of bias graph.

All 15 included RCTs were published in English, with comparable baseline characteristics between the experimental and control groups. Thirteen studies reported specific randomization methods and were rated as low risk [50,51,52,53,54,55,56,58,59,60,61,62,63], while two studies did not specify randomization [57,64]. Three studies had unclear group allocation [52,59,61], and eleven studies reported blinding. Two studies did not implement blinding and were rated as high risk [51,55], while six studies implemented double-blinding and were rated as low risk [56,57,58,62,63,64]. No studies reported allocation concealment [50,52,53,54,60,61]. All studies reported the outcomes of interest, with no evidence of data fabrication or incomplete reporting. See Figure 2 for details.

### 3.3. Short-Term Pain VAS Scores (At 4 Weeks)

#### 3.3.1. Evidence Network at 4 Weeks Post-Treatment

**Figure 3:**
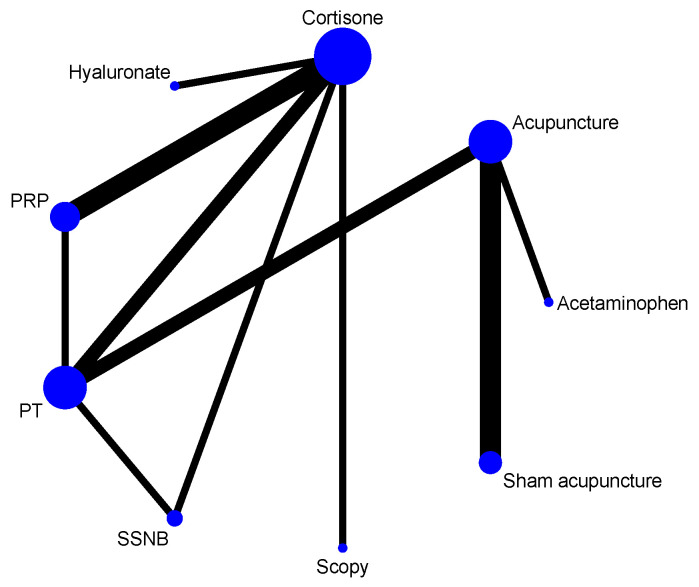
Network of treatment comparisons at 4 weeks.

Twelve RCTs [51,52,53,54,55,56,58,59,60,61,63,64] reported short-term pain VAS scores at 4 weeks post-treatment, involving nine treatment modalities. Two were three-arm clinical trials [54,59], and the remaining ten were double-arm clinical trials. The network relationship was centered on the cortisone group, forming two closed loops (Figure 3).

#### 3.3.2. Inconsistency Test at 4 Weeks Post-Treatment

**Figure 4:**
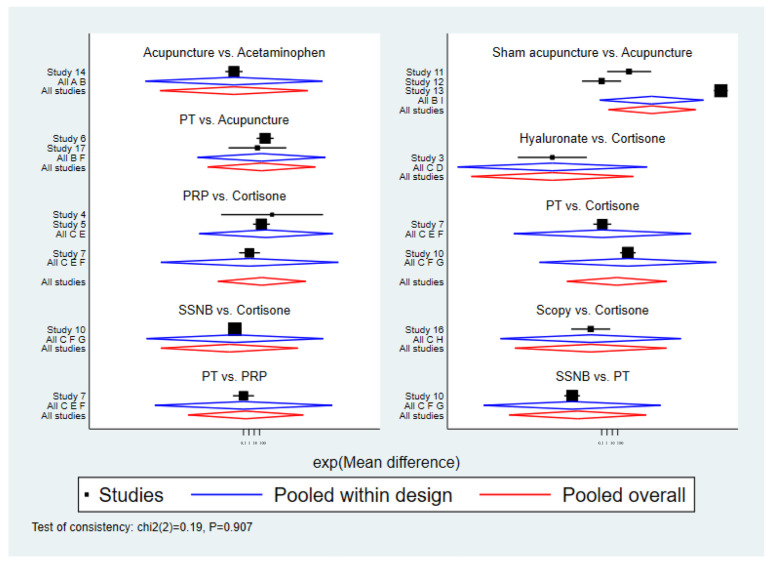
Inconsistency test for VAS scores at 4 weeks.

An inconsistency test for short-term pain at 4 weeks post-treatment was conducted using node-split models, with intergroup comparisons showing *p* > 0.05, indicating good consistency between direct and indirect comparisons (Figure 4).

#### 3.3.3. Network Meta-Analysis at 4 Weeks Post-Treatment

A network meta-analysis generated 36 pairwise comparisons. The results (Table 2) showed that acupuncture was significantly more effective than sham acupuncture in reducing VAS scores at 4 weeks post-treatment (MD: 19.39, 95% CI: 0.66–38.12, *p* < 0.05). There was no statistically significant difference in the short-term pain VAS scores among the other interventions, including acetaminophen, cortisone, hyaluronate, PRP, SSNB, and arthroscopic capsular release, 4 weeks after treatment (*p* > 0.05). The VAS was the most commonly used outcome measure for evaluating pain intensity in the included studies. In addition to VAS, some studies also utilized other assessment tools such as the Shoulder Pain and Disability Index (SPADI) and the Disabilities of the Arm, Shoulder and Hand (DASH) questionnaire to evaluate shoulder function and disability. However, due to the limited number of studies reporting these additional outcomes and the heterogeneity of the assessment tools, our network meta-analysis focused primarily on VAS scores.

#### 3.3.4. SUCRA Probability Ranking at 4 Weeks Post-Treatment

**Figure 5:**
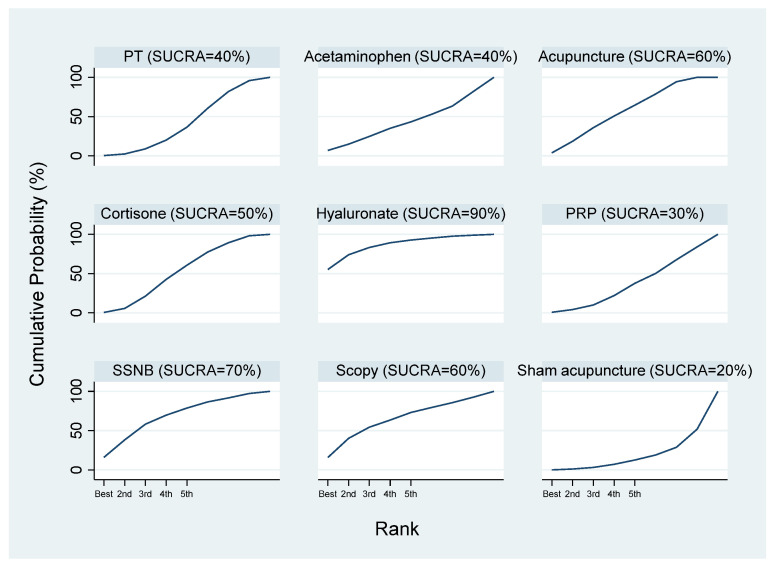
SUCRA cumulative probability ranking of VAS scores at 4 weeks.

The SUCRA results indicated that hyaluronate might be the most effective intervention, followed by SSNB, arthroscopic capsular release, acupuncture, cortisone, acetaminophen, PT, PRP, and sham acupuncture (Figure 5).

### 3.4. Long-Term Pain VAS Score at 12 Weeks Post-Treatment

#### 3.4.1. Evidence Network at 12 Weeks Post-Treatment

**Figure 6:**
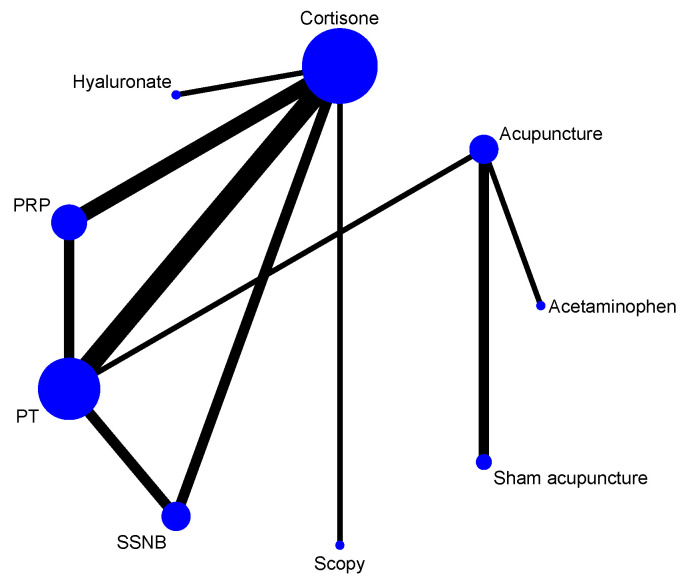
Network of treatment comparisons at 12 weeks.

Thirteen RCTs [50,52,53,54,55,56,57,58,59,60,61,63,64] reported long-term pain VAS scores at 12 weeks post-treatment, involving nine treatment modalities. Among these 13 RCTs, 3 were three-arm clinical trials [54,57,59], and the rest were double-arm clinical trials. The network relationship is centered on cortisone, which contains two closed loops (Figure 6).

#### 3.4.2. Inconsistency Test at 12 Weeks Post-Treatment

**Figure 7:**
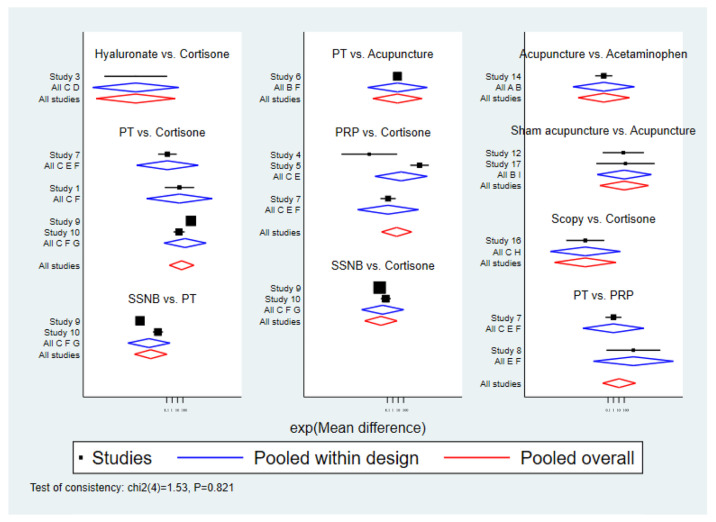
Inconsistency test of VAS scores at 12 weeks.

An inconsistency test for short-term pain at 12 weeks post-treatment was conducted using node-split models, with intergroup comparisons showing *p* > 0.05, indicating good consistency between direct and indirect comparisons (Figure 7).

#### 3.4.3. Network Meta-Analysis at 12 Weeks Post-Treatment

A network meta-analysis of long-term pain VAS scores at 12 weeks post-treatment generated 36 pairwise comparisons (Table 3). Hyaluronate was significantly more effective than PT (MD: −19.57, 95% CI: −37.23–−1.90, *p* < 0.05); SSNB was more effective than PT (MD: −9.11, 95% CI: −16.02–−2.20, *p* < 0.05); and arthroscopic capsular release was more effective than PT (MD: −16.07, 95% CI: −30.16–−1.97, *p* < 0.05). No significant differences were found among acetaminophen, cortisone, PRP, acupuncture, and sham acupuncture (*p* > 0.05).

#### 3.4.4. SUCRA Probability Ranking at 12 Weeks Post-Treatment

**Figure 8:**
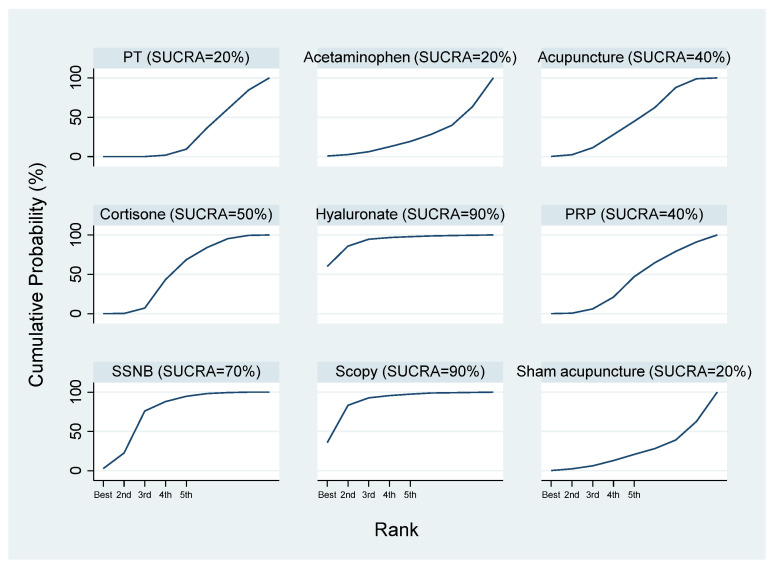
SUCRA cumulative probability ranking of VAS scores at 12 weeks.

The SUCRA results indicated that hyaluronate and arthroscopic capsular release might be the most effective interventions, followed by SSNB, cortisone, acetaminophen, PRP, PT, and sham acupuncture (Figure 8).

### 3.5. Publication Bias

**Figure 9:**
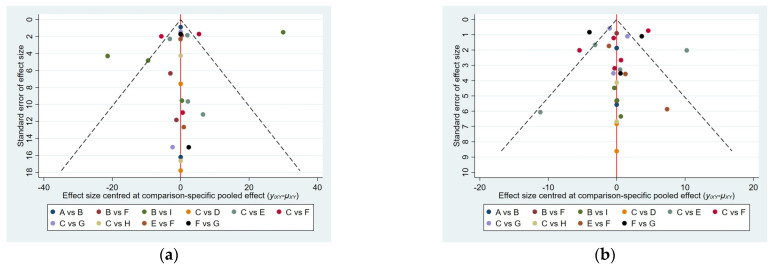
Comparison-adjusted funnel plots of publication bias for VAS scores (**a**) at 4 weeks and (**b**) 12 weeks. A, acetaminophen; B, acupuncture; C, cortisone; D, sodium hyaluronate; E, platelet-rich plasma; F, physical therapy; G, suprascapular nerve block; H, arthroscopic capsular release; I, sham acupuncture.

For the outcome indicators of the included studies with >10 participants, the funnel plot of the short-term pain VAS scores 4 weeks after treatment was reasonably symmetrical, indicating that the reporting of this outcome indicator was relatively accurate. The funnel plot for long-term pain VAS scores at 12 weeks post-treatment showed poor symmetry, suggesting possible publication bias or small sample effects (Figure 9).

## 4. Discussion

A network meta-analysis of VAS scores showed significant improvements in pain among participants in the intervention groups, indicating clinical efficacy in pain management.

With the increasing incidence of shoulder pain due to changes in lifestyle and work patterns, both Western and Chinese medical treatments have been widely used. Therefore, multiple meta-analyses and network meta-analyses have been conducted in the fields of Chinese and Western medicine [65,66,67,68,69,70,71].

A systematic review was conducted to determine the effectiveness of manual therapy directed toward the glenohumeral joint for painful shoulder conditions in improving the range of motion, pain, and shoulder functional activity and/or quality of life [65]. The evidence suggests that manual therapy interventions generally improve shoulder mobility, with high-grade/end-range mobilizations showing greater benefit. There is a trend favoring manual therapy for pain reduction, but significant heterogeneity in outcome measures and study designs limits definitive conclusions.

Eight randomized controlled trials were studied to compare manual therapy and exercise (EMT) with corticosteroid injections (CIs) for subacromial pain syndrome [66]. The results consistently showed no significant differences between EMT and CI in terms of pain reduction, functional improvement, or self-perceived recovery across all follow-up periods.

A meta-analysis was conducted to determine whether combining physiotherapy treatments offers additional benefits over exercise-only programs for shoulder pain [67]. The findings indicate that therapeutic exercise remains the cornerstone of shoulder pain management, consistently yielding significant improvements in pain and function. While adjunct therapies such as low-level and high-intensity laser therapy demonstrated moderate additional benefits, manual therapy and educational interventions provided only limited or inconsistent improvements. The heterogeneity of treatment protocols and patient populations across studies complicates direct comparisons and generalizability.

A meta-analysis was utilized to evaluate the efficacy and safety of dry needling with electrical stimulation (DNES) for musculoskeletal shoulder pain [68]. The findings suggest that DNES may provide clinically meaningful improvements in pain and disability, but these improvements are not statistically superior to those achieved with conventional physical therapy (CPT) alone.

A meta-analysis was conducted to evaluate the clinical efficacy of acupotomy in the treatment of periarthritis of the shoulder [69]. The results demonstrated that acupotomy yields superior therapeutic outcomes compared to traditional acupuncture. Nevertheless, the clinical application of acupotomy is subject to greater limitations than acupuncture, primarily due to the psychological apprehension that some patients experience toward this invasive intervention. Consequently, further rigorous evidence from clinical research is required to substantiate and compare the efficacy of acupotomy and acupuncture in clinical practice.

The meta-analytic methods were employed to investigate the efficacy of warm acupuncture combined with functional exercise in the treatment of periarthritis of the shoulder [70]. The results indicated that this combined approach was more effective in alleviating pain than other interventions. However, the relatively small sample size in this study limits the generalizability of the findings. Therefore, further clinical trials with larger sample sizes are warranted to substantiate these conclusions.

A meta-analysis protocol was proposed to evaluate the efficacy and safety of pharmacoacupuncture (PA) for frozen shoulder. PA, which combines acupuncture with herbal extract injections, is hypothesized to offer anti-inflammatory and tissue expansion effects similar to conventional corticosteroid injections and hydrodilatation [71].

Previous meta-analysis studies have focused on either Western or Chinese therapies, but few have directly compared both. This study is the first to use a network meta-analysis to compare nine types of therapies commonly used in Western and traditional Chinese medicine (acupuncture), ensuring rigor and feasibility, as well as providing direct and indirect comparisons for quantitative ranking.

The results show that acupuncture was superior to sham acupuncture in short-term pain relief at 4 weeks post-treatment, while other therapies did not show significant differences. The top five interventions for short-term pain relief were hyaluronate, SSNB, arthroscopic capsular release, acupuncture, and cortisone. For long-term pain relief at 12 weeks post-treatment, hyaluronate, SSNB, and arthroscopic capsular release appeared to show greater potential efficacy than PT. These findings should be interpreted with caution due to heterogeneity among studies and limited sample sizes. Further large-scale, multicenter studies are warranted to validate these results.

VAS scores are effective indicators of pain in patients with shoulder pain. The top three interventions for long-term pain relief were hyaluronate, SSNB, and arthroscopic capsular release, possibly due to their ability to effectively release adhesions and improve pain [52,56,57,59]. However, sensory abnormalities and psychological factors may also affect pain perception, and the VAS alone may not fully capture pain severity. Therefore, the choice of intervention should be individualized based on patient characteristics and clinical context.

Regarding the age discrepancy, particularly in the study conducted by Collinsworth et al. 2019 [55], we recognize that the mean age of approximately 20 years is atypical for frozen shoulder, which most commonly affects individuals aged 40–60 years. This difference likely reflects the inclusion of a younger population with postoperative shoulder pain or other shoulder conditions rather than classic adhesive capsulitis. Such heterogeneity in patient populations can impact the comparability of results and the generalizability of our conclusions.

This study has several limitations: (1) study quality varied, with some studies lacking blinding or adequate randomization; (2) some studies had small sample sizes, affecting the statistical power; (3) disease stage differed among participants, limiting subgroup analyses; (4) differences in participant age may have influenced treatment outcomes; (5) the exclusion of non-English studies may have introduced selection bias or language bias; and (6) the variability in the risk of bias among the included studies may have influenced the SUCRA rankings and the comparative efficacy estimates. Interventions ranked highly by the SUCRA should be interpreted with caution, as studies with a higher risk of bias may overestimate treatment effects.

## 5. Conclusions

In summary, hyaluronate, SSNB, and arthroscopic capsular release demonstrated significant advantages in the treatment of shoulder pain, with hyaluronate potentially being the optimal therapy. Clinical decisions should be individualized, and further large-scale, multicenter, high-quality RCTs are needed to confirm these findings and provide stronger evidence for clinical practice.

## Figures and Tables

**Table 1:** Study characteristics.

Studies, Year (Countries)	Age		No. of Participants		Interventions ^1^		Follow-Up /Weeks	Outcome ^2^
	T	C	T	C	T	C		
Park 2014 [50] (Korea)	56 ± 7.6	56 ± 7.6	28	25	Cortisone	PT	36	2
Wang 2015 [51] (China)	51.2 ± 7.1	49.6 ± 9.7	66	66	Acupuncture	Sham acupuncture	4.	1
Mukherjee 2017 [52] (India)	52.6 ± 7.9	48.1 ± 9.6	30	30	Cortisone	Scopy	20	1, 2
Shi 2018 [53] (China)	53.9 ± 7.7	54.0 ± 7.8	123	41	Acupuncture	Sham acupuncture	18	1, 2
Pasin 2019 [54] (Turkey)	47.73 ± 9.552	49.4 ± 9.1	30	30	Cortisone	PRP	8	1, 2
		49.86 ± 9.012		30		PT		
Collinsworth 2019 [55] (USA)	20.33 ± 1.56	20.5 ± 1.23	20	21	Acupuncture	PT	6	1, 2
Oh 2021 [56] (Korea)	52.3 ± 8.5	49.4 ± 4.9	30	30	Cortisone	Hyaluronate	24	1, 2
Parashar 2021 [57] (India)	57.20 ± 6.40	60.35 ± 5.54	20	20	SSNB	Cortisone	8	2
		57.90 ± 6.14		20		PT		
Gupta 2022 [58] (India)	46.70 ± 7.13	47.8 ± 9.56	30	30	Cortisone	PRP	24	1, 2
Mardani-Kivi 2022 [59] (Iran)	47.75 ± 10.95	48.57 ± 10.66	34	34	Cortisone	SSNB	12	1, 2
		49.20 ± 11.77		34		PT		
Somisetty 2023 [60] (India)	58.3 ± 8.1	58.5 ± 7.7	34	34	Cortisone	PRP	24	1, 2
Bağcıer 2023 [61] (Turkey)	53.3 − 5.7	53.0 − 4.9	21	21	Acupuncture	PT	12	1, 2
Karamanlioglu 2024 [62] (Turkey)	51.35 ± 8.73	53.33 ± 7.76	40	40	Acupuncture	Sham acupuncture	4	1
Cha 2024 [63] (Korea)	53.40 ± 6.51	56.63 ± 6.83	20	20	Acetaminophen	Acupuncture	8	1, 2
Ziroglu 2024 [64] (Turkey)	49.39 ± 10.95	44.25 ± 9.15	31	31	PRP	PT	8	2

^1^ PT (Physical Therapy); Scopy (arthroscopic capsular release); PRP (Platelet-Rich Plasma); Hyaluronate (Sodium Hyaluronate); SSNB (suprascapular nerve block). ^2^ 1 (Visual analogue scale at 4 week); 2 (Visual analogue scale at 8–12 week).

**Table 2:** Results of network meta-analysis for VAS scores at 4 weeks [MD (95%CI)].

Interventions	PT	Acetaminophen	Acupuncture	Cortisone	Hyaluronate	PRP	SSNB	Scopy	Sham Acupuncture
**PT**	0								
**Acetaminophen**	0.94 (−38.38, 40.27)	0							
**Acupuncture**	−5.46 (−28.64, 17.72)	−6.40 (−38.17, 25.37)	0						
**Cortisone**	−4.23 (−25.71, 17.26)	−5.17 (−49.98, 39.64)	1.23 (−30.38, 32.84)	0					
**Hyaluronate**	−27.73 (−68.69, 13.23)	−28.67 (−85.45, 28.11)	−22.27 (−69.34, 24.80)	−23.50 (−58.37, 11.37)	0				
**PRP**	1.36 (−23.48, 26.19)	0.41 (−46.10, 46.92)	6.81 (−27.16, 40.78)	5.58 (−13.34, 24.50)	29.08 (−10.60, 68.76)	0			
**SSNB**	−12.61 (−42.09, 16.88)	−13.55 (−62.70, 35.60)	−7.15 (−44.66, 30.36)	−8.38 (−37.84, 21.08)	15.12 (−30.53, 60.78)	−13.96 (−47.50, 19.58)	0		
**Scopy**	−11.23 (−50.30, 27.85)	−12.17 (−67.61, 43.27)	−5.77 (−51.20, 39.66)	−7.00 (−39.64, 25.64)	16.50 (−31.26, 64.26)	−12.58 (−50.31, 25.14)	1.38 (−42.59, 45.35)	0	
**Sham** **acupuncture**	13.93 (−15.86, 43.73)	12.99 (−23.88, 49.87)	19.39 *^1^ (0.66, 38.12)	18.16 (−18.57, 54.90)	41.66 (−8.99, 92.31)	12.58 (−26.21, 51.37)	26.54 (−15.38, 68.46)	25.16 (−23.98, 74.30)	0

*^1^: *p* < 0.05.

**Table 3:** Results of network meta-analysis for VAS scores at 12 weeks [MD (95%CI)].

Interventions	PT	Acetaminophen	Acupuncture	Cortisone	Hyaluronate	PRP	SSNB	Scopy	Sham Acupuncture
**PT**	0								
**Acetaminophen**	2.13 (−12.98, 17.24)	0							
**Acupuncture**	−2.00 (−12.44, 8.44)	−4.13 (−15.05, 6.79)	0						
**Cortisone**	−4.07 (−9.28, 1.14)	−6.20 (−22.18, 9.79)	−2.07 (−13.74, 9.60)	0					
**Hyaluronate**	−19.57 *^1^ (−37.23, −1.90)	−21.70 (−44.94, 1.55)	−17.57 (−38.09, 2.96)	−15.50 (−32.38, 1.38)	0				
**PRP**	−2.41 (−9.40, 4.57)	−4.54 (−21.19, 12.11)	−0.41 (−12.98, 12.15)	1.66 (−4.78, 8.09)	17.16 (−0.91, 35.22)	0			
**SSNB**	−9.11 *^1^ (−16.02, −2.20)	−11.24 (−27.86, 5.38)	−7.11 (−19.63, 5.41)	−5.04 (−11.94, 1.85)	10.46 (−7.78, 28.69)	−6.70 (−15.60, 2.20)	0		
**Scopy**	−16.07 *^1^ (−30.16, −1.97)	−18.20 (−38.86, 2.47)	−14.07 (−31.61, 3.47)	−12.00 (−25.09, 1.10)	3.50 (−17.86, 24.86)	−13.66 (−28.24, 0.93)	−6.96 (−21.76, 7.84)	0	
**Sham** **acupuncture**	2.59 (−12.13, 17.30)	0.46 (−14.60, 15.52)	4.59 (−5.78, 14.95)	6.65 (−8.95, 22.26)	22.15 (−0.84, 45.15)	5.00 (−11.29, 21.29)	11.70 (−4.56, 27.95)	18.65 (−1.72, 39.03)	0

*^1^: *p* < 0.05.

## Data Availability

Data sharing is not applicable. No new data were created or analyzed in this study.

## Supplementary Materials

The following supporting information can be downloaded at: https://www.mdpi.com/article/10.3390/healthcare13222920/s1.

## Abbreviations

The following abbreviations are used in this manuscript:

PT	Physical therapy
Scopy	Arthroscopic capsular release
PRP	Platelet-rich plasma
Hyaluronate	Sodium hyaluronate
SSNB	Suprascapular nerve block
