# Supplementary material for: Clinical Efficacy of Different Therapies for Painful Shoulder Conditions: A Network Meta-Analysis of Randomized Controlled Trials
Source: Healthcare (Basel). 2025 Nov 14;13(22):2920. doi: 10.3390/healthcare13222920 (PMC12652823; doi:10.3390/healthcare13222920)
Supplement: Supplementary file 1 [file healthcare-13-02920-s001.zip › healthcare-3955258-supplementary.pdf]

Supplementary Data

Supplemental Data A: Search Strategy

PubMed

Search	Query
#1	((((((((((((scapulohumeral periarthritis[MeSH Terms]) OR (frozen shoulder[MeSH Terms])) OR (adhesive periarthritis of shoulder[MeSH Terms])) OR (periarthritis of shoulder[MeSH Terms])) OR (shoulder pains[MeSH Terms])) OR (shoulder osteodystrophy[MeSH Terms])) OR (shoulder osteoarthritis[MeSH Terms])) OR (shoulder periarthritis[MeSH Terms])) OR (adhesive capsulitis of the shoulder[MeSH Terms])) OR (adhesive capsulitis[MeSH Terms])) OR (subacromial impingement syndrome[MeSH Terms])) OR (rotator cuff syndrome[MeSH Terms])) OR (rotator cuff rupture[MeSH Terms])) OR (calcific tendinitis[MeSH Terms])
#2	((((((((((proliferation therapy[MeSH Terms]) OR (prolotherapy[MeSH Terms])) OR (dextrose prolotherapy[MeSH Terms])) OR (blind glucocorticoid injection[MeSH Terms])) OR (hypertonic dextrose injection[MeSH Terms])) OR (corticosteroid injections[MeSH Terms])) OR (glucose prolotherapy[MeSH Terms])) OR (physical therapy[MeSH Terms])) OR (intra-articular injection[MeSH Terms])) OR (acupuncture[MeSH Terms])) OR (needle[MeSH Terms])
#3	(randomized controlled trial[Title/Abstract]) OR (controlled clinical trial[Title/Abstract])
#4	((#1) AND (#2)) AND (#3)

Cochrane

Search	Query
#1	(scapulohumeral periarthritis):ti,ab,kw OR (frozen shoulder):ti,ab,kw OR (adhesive periarthritis of shoulder):ti,ab,kw OR (periarthritis of shoulder):ti,ab,kw OR (shoulder pains):ti,ab,kw
#2	(shoulder osteodystrophy):ti,ab,kw OR (shoulder osteoarthritis):ti,ab,kw OR (shoulder periarthritis):ti,ab,kw OR (adhesive capsulitis of the shoulder):ti,ab,kw OR (adhesive capsulitis):ti,ab,kw
#3	(subacromial impingement syndrome):ti,ab,kw OR (rotator cuff syndrome):ti,ab,kw OR (rotator cuff rupture):ti,ab,kw OR (calcific tendinitis):ti,ab,kw
#4	(proliferation therapy):ti,ab,kw OR (prolotherapy):ti,ab,kw OR (dextrose

	prolotherapy):ti,ab,kw OR (blind glucocorticoid injection):ti,ab,kw OR (hypertonic dextrose injection):ti,ab,kw
#5	(corticosteroid injections):ti,ab,kw OR (glucose prolotherapy):ti,ab,kw OR (physical therapy):ti,ab,kw OR (intra-articular injection):ti,ab,kw OR (acupuncture):ti,ab,kw
#6	(needle):ti,ab,kw
#7	(randomized controlled trial):ti OR (controlled clinical trial):ti
#8	((#1 OR #2 OR #3) AND (#4 OR #5 OR #6) AND (#7))

Web of Sciences

Search	Query
#1	((((((((((((TS=(scapulohumeral periarthritis)) OR TS=(frozen shoulder)) OR TS=(adhesive periarthritis of shoulder)) OR TS=(periarthritis of shoulder)) OR TS=(shoulder pains)) OR TS=(shoulder osteodystrophy)) OR TS=(shoulder osteoarthritis)) OR TS=(shoulder periarthritis)) OR TS=(adhesive capsulitis of the shoulder)) OR TS=(adhesive capsulitis)) OR TS=(subacromial impingement syndrome)) OR TS=(rotator cuff syndrome)) OR TS=(rotator cuff rupture)) OR TS=(calcific tendinitis)
#2	((((((((((TS=(proliferation therapy)) OR TS=(prolotherapy)) OR TS=(dextrose prolotherapy)) OR TS=(blind glucocorticoid injection)) OR TS=(hypertonic dextrose injection)) OR TS=(corticosteroid injections)) OR TS=(glucose prolotherapy)) OR TS=(physical therapy)) OR TS=(intra-articular injection)) OR TS=(acupuncture)) OR TS=(needle)
#3	(TI=(randomized controlled trial)) OR TI=(controlled clinical trial)
#4	(#1 AND #2 AND #3)

Embase

Search	Query
#1	'scapulohumeral periarthritis'/exp OR 'scapulohumeral periarthritis' OR (scapulohumeral AND ('periarthritis'/exp OR periarthritis)) OR 'frozen shoulder':ti,ab,kw OR 'adhesive periarthritis of shoulder':ti,ab,kw OR 'periarthritis of shoulder':ti,ab,kw OR 'shoulder pains':ti,ab,kw OR 'shoulder osteodystrophy':ti,ab,kw OR 'shoulder osteoarthritis':ti,ab,kw OR 'shoulder periarthritis':ti,ab,kw OR 'adhesive capsulitis of the shoulder':ti,ab,kw OR 'adhesive capsulitis':ti,ab,kw OR 'subacromial impingement syndrome':ti,ab,kw OR 'rotator cuff syndrome':ti,ab,kw OR 'rotator cuff rupture':ti,ab,kw OR 'calcific tendinitis':ti,ab,kw

#2	'proliferation therapy'/exp OR 'proliferation therapy' OR (('proliferation'/exp OR proliferation) AND ('therapy'/exp OR therapy)) OR prolotherapy:ti,ab,kw OR 'dextrose prolotherapy':ti,ab,kw OR 'blind glucocorticoid injection':ti,ab,kw OR 'hypertonic dextrose injection':ti,ab,kw OR 'corticosteroid injections':ti,ab,kw OR 'glucose prolotherapy':ti,ab,kw OR 'physical therapy':ti,ab,kw OR 'intra-articular injection':ti,ab,kw OR acupuncture:ti,ab,kw OR needle:ti,ab,kw
#3	'randomized controlled trial':ti OR 'controlled clinical trial':ti
#4	#1 AND #2 AND #3

Supplemental Data B: PRISMA_2020_checklist

Section and Topic	Item #	Checklist item	Location where item is reported
**TITLE**			
Title	1	Identify the report as a systematic review.	Title page (lines 1–3)
**ABSTRACT**			
Abstract	2	See the PRISMA 2020 for Abstracts checklist.	Abstract (lines 10–34, page 1)
**INTRODUCTION**			
Rationale	3	Describe the rationale for the review in the context of existing knowledge.	Introduction (lines 36–88, pages 1–2)
Objectives	4	Provide an explicit statement of the objective(s) or question(s) the review addresses.	Introduction, last paragraph (lines 81–88, page 2)
**METHODS**			
Eligibility criteria	5	Specify the inclusion and exclusion criteria for the review and how studies were grouped for the syntheses.	Section 2.1. Eligibility Criteria (lines 98–109, page 3)
Information sources	6	Specify all databases, registers, websites, organisations, reference lists and other sources searched or consulted to identify studies. Specify the date when each source was last searched or consulted.	Section 2.2. Search Methods and Study Selection (lines 110–118, page 3)
Search strategy	7	Present the full search strategies for all databases, registers and websites, including any filters and limits used.	Section 2.2. Search Methods and Study Selection (lines 110–118, page 3)
Selection process	8	Specify the methods used to decide whether a study met the inclusion criteria of the review, including how many reviewers screened each record and each report retrieved, whether they worked independently, and if applicable, details of automation tools used in the process.	Section 2.3. Data Collection (lines 120–128, page 3) and Figure 1 (page 4)
Data collection process	9	Specify the methods used to collect data from reports, including how many reviewers collected data from each report, whether they worked independently,	Section 2.3. Data Collection (lines 120–128, page 3)

Section and Topic	Item #	Checklist item	Location where item is reported
		any processes for obtaining or confirming data from study investigators, and if applicable, details of automation tools used in the process.	
Data items	10a	List and define all outcomes for which data were sought. Specify whether all results that were compatible with each outcome domain in each study were sought (e.g. for all measures, time points, analyses), and if not, the methods used to decide which results to collect.	Section 2.3. Data Collection (lines 120–128, page 3); Table 1 (page 4)
	10b	List and define all other variables for which data were sought (e.g. participant and intervention characteristics, funding sources). Describe any assumptions made about any missing or unclear information.	Section 2.3. Data Collection (lines 120–128, page 3); Table 1 (page 4)
Study risk of bias assessment	11	Specify the methods used to assess risk of bias in the included studies, including details of the tool(s) used, how many reviewers assessed each study and whether they worked independently, and if applicable, details of automation tools used in the process.	Section 2.4. Risk of Bias Assessment (lines 129–131, page 3); Figure 2 (page 5)
Effect measures	12	Specify for each outcome the effect measure(s) (e.g. risk ratio, mean difference) used in the synthesis or presentation of results.	Section 2.5. Data Synthesis and Analysis (lines 132–143, page 3)
Synthesis methods	13a	Describe the processes used to decide which studies were eligible for each synthesis (e.g. tabulating the study intervention characteristics and comparing against the planned groups for each synthesis (item #5)).	Section 2.5. Data Synthesis and Analysis (lines 132–143, page 3); Results (pages 4–10)
	13b	Describe any methods required to prepare the data for presentation or synthesis, such as handling of missing summary statistics, or data conversions.	Section 2.5. Data Synthesis and Analysis (lines 132–143, page 3); Results (pages 4–10)
	13c	Describe any methods used to tabulate or visually display results of individual studies and syntheses.	Section 2.5. Data Synthesis and Analysis (lines 132–143, page 3); Results (pages 4–10)
	13d	Describe any methods used to synthesize results and provide a rationale for the choice(s). If meta-analysis was performed, describe the model(s), method(s) to identify the presence and extent of statistical heterogeneity, and software package(s) used.	Section 2.5. Data Synthesis and Analysis (lines 132–143, page 3); Results (pages 4–10)

Section and Topic	Item #	Checklist item	Location where item is reported
	13e	Describe any methods used to explore possible causes of heterogeneity among study results (e.g. subgroup analysis, meta-regression).	Section 2.5. Data Synthesis and Analysis (lines 132–143, page 3); Results (pages 4–10)
	13f	Describe any sensitivity analyses conducted to assess robustness of the synthesized results.	Section 2.5. Data Synthesis and Analysis (lines 132–143, page 3); Results (pages 4–10)
Reporting bias assessment	14	Describe any methods used to assess risk of bias due to missing results in a synthesis (arising from reporting biases).	Section 3.5. Publication Bias (lines 228–233, page 10); Figure 9 (page 10)
Certainty assessment	15	Describe any methods used to assess certainty (or confidence) in the body of evidence for an outcome.	Not applicable
**RESULTS**			
Study selection	16a	Describe the results of the search and selection process, from the number of records identified in the search to the number of studies included in the review, ideally using a flow diagram.	Section 3.1. Literature Search Results (lines 145–153, page 4); Figure 1 (page 4)
	16b	Cite studies that might appear to meet the inclusion criteria, but which were excluded, and explain why they were excluded.	Section 3.1. Literature Search Results (lines 145–153, page 4)
Study characteristics	17	Cite each included study and present its characteristics.	Table 1 (page 4–5)
Risk of bias in studies	18	Present assessments of risk of bias for each included study.	Section 3.2. Risk of Bias Assessment (lines 156–166, page 5); Figure 2 (page 5)
Results of individual studies	19	For all outcomes, present, for each study: (a) summary statistics for each group (where appropriate) and (b) an effect estimate and its precision (e.g. confidence/credible interval), ideally using structured tables or plots.	Table 2 (page 7), Table 3 (page 9)
Results of syntheses	20a	For each synthesis, briefly summarise the characteristics and risk of bias among contributing studies.	Section 3.3–3.4 (pages 6–10); Figures 3–8 (pages 6–10)
	20b	Present results of all statistical syntheses conducted. If meta-analysis was done, present for each the summary estimate and its precision (e.g.	Section 3.3–3.4 (pages 6–10); Figures 3–8 (pages 6–10)

Section and Topic	Item #	Checklist item	Location where item is reported
		confidence/credible interval) and measures of statistical heterogeneity. If comparing groups, describe the direction of the effect.	
	20c	Present results of all investigations of possible causes of heterogeneity among study results.	Section 3.3–3.4 (pages 6–10); Figures 3–8 (pages 6–10)
	20d	Present results of all sensitivity analyses conducted to assess the robustness of the synthesized results.	Section 3.3–3.4 (pages 6–10); Figures 3–8 (pages 6–10)
Reporting biases	21	Present assessments of risk of bias due to missing results (arising from reporting biases) for each synthesis assessed.	Section 3.5. Publication Bias (lines 228–233, page 10); Figure 9 (page 10)
Certainty of evidence	22	Present assessments of certainty (or confidence) in the body of evidence for each outcome assessed.	Not applicable
**DISCUSSION**			
Discussion	23a	Provide a general interpretation of the results in the context of other evidence.	Section 4. Discussion (lines 240–297, pages 10–12)
	23b	Discuss any limitations of the evidence included in the review.	Section 4. Discussion (lines 320–328, page 12)
	23c	Discuss any limitations of the review processes used.	Section 4. Discussion (lines 320–328, page 12)
	23d	Discuss implications of the results for practice, policy, and future research.	Section 4. Discussion (lines 329–334, page 12)
**OTHER INFORMATION**			
Registration and protocol	24a	Provide registration information for the review, including register name and registration number, or state that the review was not registered.	Register name: Sui-Yi Guo<br>Registration number: 10.17605/OSF.IO/VQC6W
	24b	Indicate where the review protocol can be accessed, or state that a protocol was not prepared.	A review protocol already exists, located at https://osf.io/VQC6W/overview.
	24c	Describe and explain any amendments to information provided at registration or in the protocol.	No amendments
Support	25	Describe sources of financial or non-financial support for the review, and the role of the funders or sponsors in the review.	Non-financial support for the review.

Section and Topic	Item #	Checklist item	Location where item is reported
Competing interests	26	Declare any competing interests of review authors.	No conflicts of interest.
Availability of data, code and other materials	27	Report which of the following are publicly available and where they can be found: template data collection forms; data extracted from included studies; data used for all analyses; analytic code; any other materials used in the review.	Data Availability Statement (lines 287-288)
